# Development of a community-based model for respiratory care services

**DOI:** 10.1186/1472-6963-12-193

**Published:** 2012-07-09

**Authors:** Emily J Henderson, Greg P Rubin

**Affiliations:** 1 Evaluation, Research and Development Unit, Durham University, School of Medicine and Health, Wolfson Research Institute, Stockton-on-Tees, UK

**Keywords:** Chronic care, Respiratory diseases, Delphi consensus

## Abstract

**Background:**

Chronic respiratory diseases are a major cause of mortality and morbidity, and represent a high chronic disease burden, which is expected to rise between now and 2020. Care for chronic diseases is increasingly located in community settings for reasons of efficiency and patient preference, though what services should be offered and where is contested. Our aim was to identify the key characteristics of a community-based service for chronic respiratory disease to help inform NHS commissioning decisions.

**Methods:**

We used the Delphi method of consensus development. We derived components from Wagner’s Chronic Care Model (CCM), an evidence-based, multi-dimensional framework for improving chronic illness care. We used the linked Assessment of Chronic Illness Care to derive standards for each component.

We established a purposeful panel of experts to form the Delphi group. This was multidisciplinary and included national and international experts in the field, as well as local health professionals involved in the delivery of respiratory services. Consensus was defined in terms of medians and means. Participants were able to propose new components in round one.

**Results:**

Twenty-one experts were invited to participate, and 18 agreed to take part (85.7% response). Sixteen responded to the first round (88.9%), 14 to the second round (77.8%) and 13 to the third round (72.2%). The panel rated twelve of the original fifteen components of the CCM to be a high priority for community-based respiratory care model, with varying levels of consensus. Where consensus was achieved, there was agreement that the component should be delivered to an advanced standard. Four additional components were identified, all of which would be categorised as part of delivery system design.

**Conclusions:**

This consensus development process confirmed the validity of the CCM as a basis for a community-based respiratory care service and identified a small number of additional components. Our approach has the potential to be applied to service redesign for other chronic conditions.

## Background

There is increasing demand on health services in the UK and other Western countries as a result of ageing populations and the rising prevalence of chronic disease. The current structure of chronic care, which is derived from models of hospital-based acute care, is in need of reform in order to address the specific care needs of long term illness [1,2]. In response to this need, recent reforms to health and social care in the UK have focused on increasing community-based care [3,4], including patient self-care through an Expert Patient Programme [5-7], which allows for personalised services and care closer to home.

Chronic respiratory diseases represent a considerable burden on health care services. They contribute to social inequalities in life expectancy, notably through preventable early deaths, as well as to excess winter deaths [8]. Chronic obstructive pulmonary disease (COPD) and asthma, and are major causes of morbidity [9,10]. In the UK, COPD alone accounts for 1.4 million GP consultations per year and 1 in 8 emergency admissions [11], and its prevalence is expected to rise between 2010 and 2020 [8].

There is evidence for the effectiveness of a range of services for COPD when provided in community settings. Pulmonary rehabilitation is a safe and clinically effective intervention, and in less severe cases it can be delivered at home as an alternative to outpatient care [12-16]. It improves exercise capacity, health status and health-related quality of life [12-17]; reduces hospital admissions, time spent in hospital and mortality [13,17]. Hospital at home is safe, reduces the need for emergency services, and improves quality of life and self-management [18,19]. It is as effective as inpatient care in terms of mortality and hospital readmissions, though COPD patients may prefer inpatient services to hospital at home [20,21]. Self-management education improves self-efficacy and self-care, and can reduce hospital admissions [22,23]. Action plans in particular improve self-management knowledge, such as patients’ ability to recognise and respond appropriately to exacerbations [24]. Group therapy improves health knowledge and quality of life [25]. Multiple intervention programmes reduce hospital admissions and improve quality of life, but require multi-disciplinary input [1,26]. They are effective in patients with exercise impairment and to reduce hospital admissions and readmission [27,28].

Existing theoretical models of service provision for chronic disorders include the case management model [29], the complex adaptive chronic care model [30] and psychosocial models [31], though the CCM is the most widely accepted because it is the most comprehensive [32]. However, a systematic review found a limited number of studies that evaluated the effectiveness of the CCM components in COPD management [33]. In the north east of England, primary care commissioners and secondary care providers are collaborating to reduce inefficiencies and to improve quality of care through Quality Improvement Plans [34]. At their request, we sought to identify the key characteristics of a community-based service for chronic respiratory diseases. We defined community-based care to include services that are offered either 1) outside of a family practice or hospital setting, for example in the patient’s home, or 2) on premises from which family practice or hospital services are delivered, but which are outwith what is normally offered.

## Methods

### Aim

To develop consensus among professionals involved in the care of chronic respiratory diseases on the key characteristics of a community-based respiratory service.

### Materials

To develop a model of the ideal service, we used the Delphi technique of consensus development [35]. This involves the generation of group judgements and allows opportunities for participants to revise their responses after formal feedback of group views. We developed a modified Delphi survey based on model elements of Wagner’s Chronic Care Model (CCM). The CCM is an evidence-based multi-dimensional framework for improving chronic illness care, and includes six model elements: organisation of health care; self-management support; delivery system design; decision support; clinical information systems; and community resources and policies [36]. Together these are theorised to lead to productive interactions between the informed, activated patient and the prepared, proactive practice team.

We examined the elements described by both the CCM areas and the linked Assessment of Chronic Illness Care (v3.5) [37,38]. We identified 15 primary components represented by five of the six model elements (Table 1). We determined that organisation of health care, the sixth model element, was beyond the scope of this exercise since it focussed on chronic care at the level of the health service. The standard to which the components should be delivered was derived from the Assessment of Chronic Illness Care survey [38]. We also offered respondents the opportunity to propose additional components in the course of Round 1.

**Table 1:** Components of a community-based respiratory service and their associated model elements, adapted from Wagner’s Chronic Care Model^*^

**Model Elements**	**Components**
Community resources and policy	Links with community services and resources
Self-management support	Carer and family support
	Patient behaviour-change interventions, e.g. pulmonary rehabilitation
	Self-management support strategies
Delivery system design	Regular patient follow-up
	Care provided by a multi-disciplinary team
	Integration of care between primary and secondary sectors
	Integrating palliative care into the community
Decision Support	Evidence based guidelines
	Involving specialists to improve the care delivered by the team
	Continuing professional development and advanced training for the team
Clinical information systems	Individual patient care/treatment plans
	Disease registers of COPD patients
	Performance monitoring of the COPD team
	Identifying relevant subgroups of patients for care

*sourced from Improving Chronic Illness Care [37,38].

### Sample

We established a purposeful sample by inviting 21 experts [39] to take part, aiming to reach the recommended sample size of around 12 participants and anticipating that response would diminish over consecutive rounds [35]. The panel was multidisciplinary and comprised national and international experts in the field, as well as health professionals involved in the local delivery of respiratory services.

### Design

**Figure 1:**
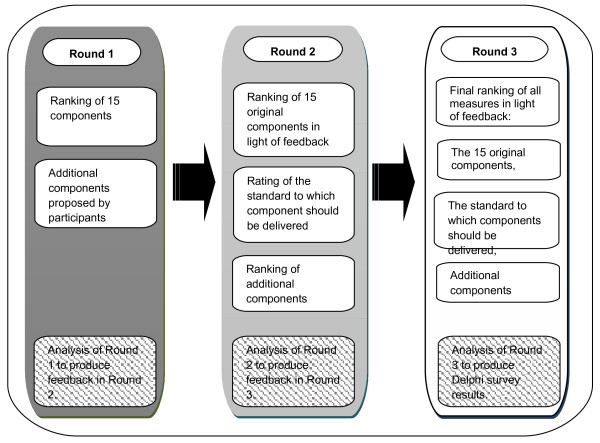
Procedure of the series of rounds for ranking and weighting of components.

Three rounds were held to build consensus. Surveys were distributed and returned by email. One reminder was sent to non-respondents for each round (Figure 1). Participants who did not respond in round two were nevertheless encouraged and allowed to respond in round three [40].

### Analysis

The panel rated the importance of each component on a 9-point scale and the results were grouped by level of priority as either low (1–3 points), moderate (4–6 points) or high (7–9 points) [41].

Consensus was developed by a two step process. First, for any component to reach consensus, the group median and interquartile range had to fall within one level of priority only (either low, moderate or high). Second, the extent to which consensus was met—either general, full or pure—is based on the group mean, standard deviations and the presence of outliers (Table 2).

**Table 2:** Definition of the three increasing levels of consensus

General consensus	1. Median and interquartile range fall within one priority level only
	2. Mean standard deviation extends beyond one level of the scale and outliers are present.
Full consensus	1. Median and interquartile range fall within one priority level only
	2. Mean standard deviation extends beyond one level of the scale but no outliers are present.
Pure consensus	1. Median and interquartile range fall within one priority level only
	2. Mean standard deviation lies within one level of the scale and no outliers are present.

For the standards to which each component should be delivered, we used a 4-point Likert scale. Four point scales have previously been shown to produce stable findings in Delphi studies [42]. Consensus was reached when the interquartile range lay within 1 unit of the median (on a 4-point Likert scale, with 1 being lowest standard and 4 being the highest).

### Ethical issues

No ethical issues were identified in this consensus study, which involved only email contact with health professionals, and ethical approval was not sought.

## Results

### Respondents

We received expressions of interest to participate from 18 of the 21 experts invited (85.7% response) (Table 3). Sixteen provided responses to the first round (88.9%), 14 to the second round (77.8%) and 13 to the third round (72.2%). Most individuals provided responses to all rounds, although there were 5 people who did not, as we allowed any non-respondents to reply to subsequent rounds [40].

**Table 3:** Professions represented in the sample by Delphi round

**Invited (n)**	**Round 1**	**Round 2**	**Round 3**
	**n**	**n**	**n**
Consultant respiratory physician (2)	2	2	2
Respiratory specialist (4)	4	3	2
Respiratory physiology lead (1)	0	0	0
General practitioner (3)	3	2	3
Primary care academic (3)	2	3	3
Respiratory specialist nurse (2)	1	0	0
Community respiratory nurse (1)	0	0	0
Practice nurse (1)	1	1	0
Commissioning lead (2)	2	2	2
Practice based commissioning manager (1)	1	1	1
Team manager (1)	0	0	0
Total	16	14	13

### The survey

Over the three rounds, the priority ratings for all components either lowered or remained the same, and there was a narrowing of interquartile ranges. Of the original fifteen components, three were defined as having met ‘general consensus’; five were considered to have reached ‘full consensus’; and four components reached ‘pure consensus’. All those reaching consensus were considered to be a high priority (rating 7–9) for the model. The standard of delivery for each component was also high, in most cases being 1 on a scale of 1 to 4.

Twelve additional components were generated by participants during the first round (Table 4). Consensus was less likely for these. Only four components were considered to be a high priority for the model, with one reaching ‘general consensus’ and three reaching ‘full consensus’. We assigned each new component to a model element.

**Table 4:** Additional component that arose from Round One

**Model Elements**	**Additional Components**
Community resources and policy	Fuel poverty
	Lifestyle
	Air pollution
Self-management support	Telephone helpline
	Smoking cessation
Delivery system design	Transport is available to the place of delivery
	A range of community locations
	Delivery in the patient home
	End of life care
	Long term oxygen therapy
	Acute exacerbations
	Rapid access to diagnostics
Decision support	n/a
Clinical information systems	n/a

### Final components

There was consensus for twelve of the original fifteen components and four of the additional components (Table 5). In all cases where consensus was met, the component was considered by respondents to be a high priority for the model.

**Table 5:** Model of community-based respiratory services

	**Type of consensus met**	**Component**	**Standard to which component should be met**	**Level of priority**
**Original components**	**General**	Integrating care between primary and secondary sectors	A high priority and all chronic disease interventions include active coordination between primary care, specialists and other relevant groups.	High
		Performance monitoring of the COPD team	Is timely, specific to the team, routine and personally delivered by a respected opinion leader to improve team performance.	High
		Individual patient care/treatment plans	Established collaboratively and include self management as well as clinical management. Follow-up occurs and guides care at every point of service.	High
	**Full**	Continuing professional development and advanced training for the team	Include training in all practice teams in chronic illness care methods such as population-based management, and self-management support.	High
		Integrating palliative care into the community	N/A	High
		Links with community services and resources	Actively sought to develop formal supportive programs in order and policies across the entire system.	High
		Carer and family support	An integral part of care and includes systematic assessment and routine involvement in peer support, groups or mentoring programs.	High
		Self-management support strategies	Provided by trained clinical educators who are designed to do self-management support, affiliated with each practice, and who see patients on referral.	High
	**Pure**	Evidence based guidelines	Available, supported by provider education and integrated into care through reminders and other proven provider behaviour	High
		Care provided by a multi-disciplinary team	Assured by regular team meetings to address guidelines, roles and accountability, and problems in chronic illness care.	High
		Disease registers of COPD patients	Tied to guidelines which provide prompts and reminders about needed services.	High
		Patient behaviour-change interventions, e.g. pulmonary rehabilitation	Readily available and an integral part of routine care.	High
**Additional components**	**General**	Acute exacerbation	N/A	High
	**Full**	Smoking cessation	N/A	High
		End-of-life care	N/A	High
		Long-term oxygen therapy	N/A	High

N/A - not available.

## Discussion

### Summary of main findings

Using a three-round Delphi consensus method, we have identified the key components of a community-based respiratory service and the standard to which they should be delivered. We used the CCM as our theoretical model and practical guide. Twelve of the fifteen original components of the CCM were considered to be a high priority for community-based respiratory care. Respondents considered that all the agreed components should be delivered to a high standard. We also identified four components additional to the CCM. These may be relevant to chronic care for other diseases as well as for COPD.

### Strengths and limitations of the study

A range of disciplines was represented, with local, national and international expertise. Delphi consensus methods do not involve face-to-face discussions between respondents, but do allow for bringing together opinions of people from a range of geographic backgrounds or busy professionals who might not otherwise have the time to meet for a day of discussion [41]. Not all participants responded to all three rounds, which is an expected feature for consensus development techniques. The response rate for each round of the study was, however, greater than the recommended minimum of 70% [43]. There was a low response from nurses, who comprised nearly one quarter of all professionals invited to participate, but represented under one eighth of the final sample. However, given the mix of professionals involved in the delivery of community-based respiratory services in the UK, we assert the sample was representative of the range of professionals in the UK.

### Comparison with existing literature

Five of the six model elements from the CCM were represented in the final consensus: self-management support; delivery system design; decision support; clinical information systems; and community resources and policies [36]. This confirms the face validity of the CCM as a model for community-based chronic care. Three of the twelve additional components that arose from round one met with full consensus, and one reached general consensus. All four can be categorised as new components of delivery system design. One (smoking cessation) could be considered as a behaviour change intervention, a second (long term oxygen therapy) is a specific therapeutic intervention, and the third (acute exacerbation) refers to responsiveness of the health system. We have taken the fourth, end-of-life care, to be wider than the CCM component for integrating palliative care into the community. As such, it represents a new and distinct component of delivery system design.

In their systematic review, Dennis et al. [44] found four of the six model elements of the CCM to be effective in disease management. These were continuing professional development for the multidisciplinary team (the decision support element); clear roles of responsibility in a system where self-management is not embedded in primary care (the delivery system design element); and disease registers (the clinical information system element) to facilitate decision support (the decision support element). Little evidence was found for the model elements of community resources and policies, and organisation of health care in primary care. This supports our decision not to include the latter, though we did find a clear need from practitioners for the former. Our findings are in keeping with those of Dennis et al. However, we chose not to address organisation of health care in primary care, which they considered important though lacking in supporting evidence. Furthermore, Adams et al. [33] do not treat this as a separate model element in their analysis of the CCM in COPD.

### Implications for practice

The high standard to which most components were recommended to be delivered could pose a challenge to implementation. They may be better viewed as a goal to which service providers could be required to work. Some of the recommended components are not routinely addressed or provided, either in primary or secondary care, and their provision will have resource implications. This approach to developing a model of care for COPD, building upon a well validated conceptual model of care, is applicable to other chronic conditions and consideration should be given to using it to inform the commissioning of new models of chronic care.

Table 6, derived from the Adams et al. [33] systematic review of interventions applying the CCM in COPD care, summarises the four model elements of the CCM that each study applied to their model of community-based care. The review dichotomised findings by either one element or multiple elements. It found significantly lower rates of hospitalizations and emergency/unscheduled visits and a shorter length of stay where multiple main elements were applied. Only half of the 18 community-based studies reviewed included two or more of the model elements of the CCM, and only two included all.

**Table 6:** Components of the Chronic Care Model that studies applied in the delivery of community-based services*

**No. of components**		**Self- management**	**Delivery system design**	**Decision support**	**Clinical information systems**
1	Farrero et al. 2001		X		
	Weinberger et al. 2002	X			
	Brough et al. 1982; Cockcroft et al. 1987; Howland et al. 1986; Littlejohns et al. 1991 Zimmerman et al. 1996	X			
	Goransson et al. 2003; Emery et al. 1998	X			
2	Steinel and Madigan 2003		X	X	
	Haggerty et al. 1991; Hermiz et al. 2002; Hernandez et al. 2003	X	X		
	Monninkhof et al. 2003	X	X		
3	Bourbeau et al. 2003; Neff et al. 2003	X	X	X	
4	Barnett 2003	X	X	X	X
	Rea et al. 2004	X	X	X	X

*Adapted from Adams et al. 2007 [33].

Table 7, also based on Adams et al., summarises the combination of each of the four model elements used to measure improvements in service delivery of COPD. No measures studied used a combination all four model elements, save hospitalisation, making it difficult to draw conclusions as to which combination is optimal. The self-management model element features in most studies that show significant results for a measure of improvement, while the clinical information system model element is rarely used. Dyspnoea, hospitalisation and length of stay seem to require use of multiple model elements. Knowledge may be improved by self-management alone. Mortality does not appear to be improved by any combination, and performance and lung function are understudied in community-based settings.

**Table 7:** Measures of improvement in COPD community-based services*

	**No. of model elements**				**Successful combinations**	
	**1**	**2**	**3**	**4**	**No.**	**Model Element**
**Knowledge**	2 sig (Emery et al. 1998; Goransson et al. 2003) 2 n/s (Brough et al. 1982; Cockcroft et al. 1987)	2 sig (Hermiz et al. 2002; Hernandez et al. 2003)	n/a	n/a	1	Self-management
					2	Self-management
						Delivery system design
**Dyspnoea**	1 n/s (Zimmerman et al. 1996)	2 n/s (Hermiz et al. 2002; Monninkhof et al. 2003)	1 sig (Neff et al. 2003) 1 n/s (Bourbeau et al 2003)	n/a	3	Self-management
						Delivery system design
						Decision support
**Quality of life**	4 n/s (Emery et al. 1998; Weinberger et al. 2002; Cockcroft et al. 1987; Littlejohns et al. 1991)	2 n/s (Hermiz et al. 2002; Monninkhof et al. 2003)	1 sig (Neff et al. 2003) 1 n/s (Bourbeau et al. 2003)	n/a	3	Self-management
						Delivery system design
						Decision support
**Lung function**	n/a	n/a	n/a	1 sig (Rea et al. 2004)	4	Self-management
						Delivery system design
						Decision support
						Clinical information system
**Performance**	n/a	n/a	n/a	n/a	n/a	n/a
**Mortality**	3 n/s (Farrero et al. 2001; Cockcroft et al. 1987; Littlejohns et al. 1991)	3 n/s (Monninkhof et al. 2003; Hernamdez et al. 2002)	n/a	1 n/s (Rea et al. 2004)	n/a	n/a
**Health care use**	n/a	4 sig (Hermiz et al. 2002; Hernandez et al. 2002; Steinel and Madigan 2003; Haggarty et al. 1991)	2 sig (Neff et al. 2003; Bourbeau et al. 2003)	n/a	2	Self-management
						Delivery system design
					2	Delivery system design
						Decision support
					3	Self-management
						Delivery system design
						Decision support
**Hospitalisation**	1 sig (Farrero et al. 2001) 3 n/s (Cockcroft et al. 1987; Littlejohns et al. 1991; Weinberger et al. 2002)	4 sig (Hermiz et al. 2002; Hernandez et al. 2003; Steinel and Madigan 2003; Haggerty et al. 1991)	2 sig (Bourbeau et al. 2003; Neff et al. 2003)	2 sig (Barnett 2003; Rea et al. 2004)	1	Self-management
					2	Self-management
						Delivery system design
					2	Delivery system design
						Decision support
					3	Self-management
						Delivery system design
						Decision support
					4	Self-management
						Delivery system design
						Decision support
						Clinical information systems
**Length of stay**	3 n/s (Cockcroft et al. 1987; Littlejohns et al. 1991; Farrero et al. 2001)	2 sig (Hernandez et al. 2002; Steinel and Madigan 2003)	2 sig (Bourbeau et al. 2003; Neff et al. 2003)	n/a	2	Self-management
						Delivery system design
					2	Delivery system design
						Decision support
					3	Self-management
						Delivery system design
						Decision support

*Adapted from Adams et al. 2007 [33].

## Conclusions

In this development of a model for community-based respiratory services, consensus was reached on the inclusion of a large proportion of components derived from a well-accepted theoretical model (the CCM). We generated a small number of additional components and these may have wider relevance in chronic care.

This approach to developing consensus has the potential to be applied to service redesign for other chronic conditions. It is likely to be most relevant where a range of professionals provide care for the condition in question and where their experience and the setting for that care is not highly compartmentalised. Examples of amenable conditions could include rheumatological disorders and diabetes.

The number of components that were agreed to be necessary to community respiratory care, and the high standard to which they should be delivered, may pose a challenge to implementation. In the UK, for example, they may need to be seen as an aspiration to which commissioners would work within the context of current resource limitations.

## Abbreviations

CCM, Chronic care model; NHS, National health service; PCT, Primary care trust.

## Competing interests

The authors declare that they have no competing interests.

## Authors’ contributions

EH and GR designed the study analysed the results. EH collected the data. EH and GR prepared, read and approved the final manuscript.

## Authors’ information

EH is a researcher for and GR is director of the Evaluation, Research and Development Unit, which provides independent health services research in primary care.

## Pre-publication history

The pre-publication history for this paper can be accessed here:

http://www.biomedcentral.com/1472-6963/12/193/prepub
